# Rare and Occult Müllerian Deformity Diagnosed Intraoperatively During Lower Segment Caesarean Section

**DOI:** 10.7759/cureus.46861

**Published:** 2023-10-11

**Authors:** Prakher Shrivastava, Deepti Shrivastava, Priyal Shrivastava, Shobha Rawlani, Vilas Chimurkar

**Affiliations:** 1 Medicine, Jawaharlal Nehru Medical College, Datta Meghe Institute of Higher Education and Research, Wardha, IND; 2 Obstetrics and Gynecology, Jawaharlal Nehru Medical College, Datta Meghe Institute of Higher Education and Research, Wardha, IND; 3 Radiology, Jawaharlal Nehru Medical College, Datta Meghe Institute of Higher Education and Research, Wardha, IND; 4 Anatomy, Jawaharlal Nehru Medical College, Datta Meghe Institute of Higher Education and Research, Wardha, IND

**Keywords:** rare, uuwrh, agenesis, unicornuate, mda

## Abstract

Uterine anomalies are the rare entities seen due to fusion defects or agenesis of Müllerian ducts in embryonic life. They are usually associated with renal defects. If non-obstructive in nature, they hardly become symptomatic until the beginning of their obstetric carrier, when they present with infertility, recurrent pregnancy loss, preterm pregnancy, rudimentary horn rupture, an ectopic baby, and numerous more negative effects. We are presenting a case report of a patient who was suffering from infertility, attempted IUI treatment five times, then stopped the treatment due to economic affordability issues, conceived spontaneously, and underwent preterm lower segment cesarean section (LSCS) due to fetal distress. During LSCS, it was found to be type 2C of the American Fertility Society (AFS) Müllerian anomaly. The post-operative period was uneventful; both mother and baby were fine, and they were discharged.

## Introduction

Müllerian duct malformations are the result of incomplete or poor embryonic development, fusion defect, or complete agenesis of Müllerian ducts. Etiopathogenesis of these anomalies is multifactorial and may be due to teratogen exposure in early embryonic life like category C and D drugs, radiations, toxoplasmosis, rubella cytomegalovirus, herpes simplex, and HIV (TORCH) group of infection, acute viral illness, or genetic change, in particular, the B-cell lymphoma 2 (BCL2) mutation. 

Müllerian anomalies occur in 0.2%-3.8% of fertile women and 3.5%-6.3% of infertile women. In one study, the malformations with the highest prevalence were uterine septum at 4.4% (~20%) [1,2]. One of the rarest prevalent Müllerian duct anomalies (MDAs) is a didelphus uterus, also referred to as a double uterus. The paramesonephric ducts’ failure to fuse in a specific location or along their regular line of fusion causes uterine duplication. Individual horns are completely mature, regular in dimensions, and have two cervices in each didelphus. There is only one fallopian tube per uterus. Some patients have primary infertility manifestations, while others are unaffected [3]. Normal pregnancy can only happen in a small percentage of women, although obstetric difficulties such as missed abortion, fetal death, premature birth, and malpresentation are common. Ignored bicornuate and septate uteruses also have stint pregnancy outcomes, with term delivery rates of only 40% and term delivery rates of only 45% for unicornuate and didelphus uteruses, respectively [4]. 

Severe gynecologic and obstetric problems, like infertility, endometriosis, and pregnancy termination, are usually linked to MDAs. In as many as 30 to 50% of cases, they are also frequently accompanied by renal abnormalities. Renal agenesis, abnormal kidney, hypoplastic, fusion, replication, and malrotation of the kidneys are among the frequent renal defects [4,5]. Thus, during the examination, it is crucial to identify both kidneys. Women who are born with complicated anorectal abnormalities typically also have MDAs. Cardiovascular abnormalities (14.5%), spinal anomalies (29%) (i.e., fused or wedged vertebral bodies, spina bifida; 22-23%), and syndromes such the Klippel-Feil syndrome (7%) are additional birth defects that are frequently linked to MDAs [6]. Other conditions such as renal or cardiac anomalies in patients with MDAs may also have an impact on their fertility, modes of delivery, and obstetrical outcomes. These conditions include those involving vertebral imperfections, anal atresia, cardiovascular deficiencies, tracheoesophageal fistula, kidney abnormalities, and limb abnormalities (VACTERL association).

## Case presentation

A 31-year-old female presented with primary infertility for seven years. On sonosalpingography, the patency of at least one tube was confirmed, as the free flow of normal saline was observed in the pouch of Douglas. Her anti-Müllerian hormone (AMH) was 2.1 ng/mL, and her antral follicular count was good. After three cycles of ovulation induction and follicular monitoring, she underwent intrauterine insemination four times without any success. The husband’s semen parameters were within normal limits. Due to personal and economic reasons, she discontinued further treatment. 

**Figure 1:**
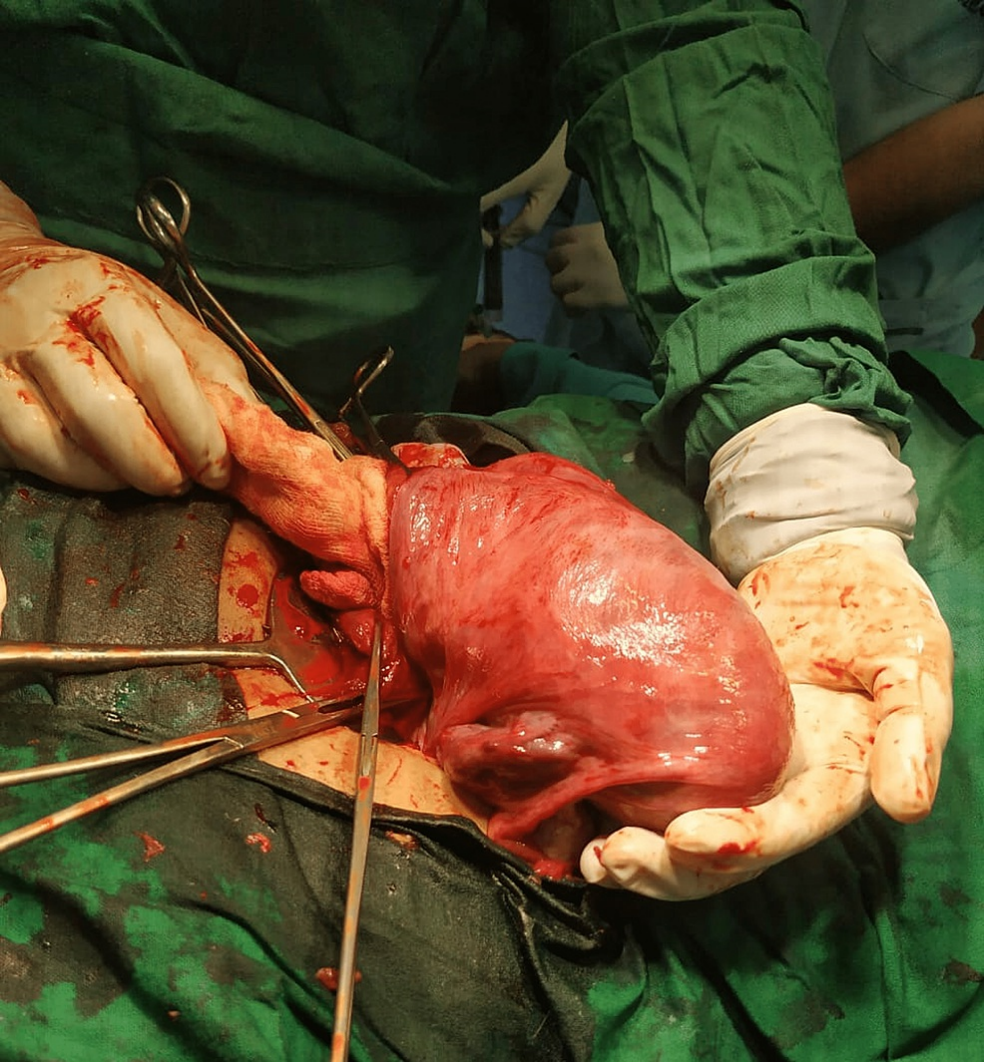
Banana-shaped unicornuate uterus Photo credit: author

**Figure 2:**
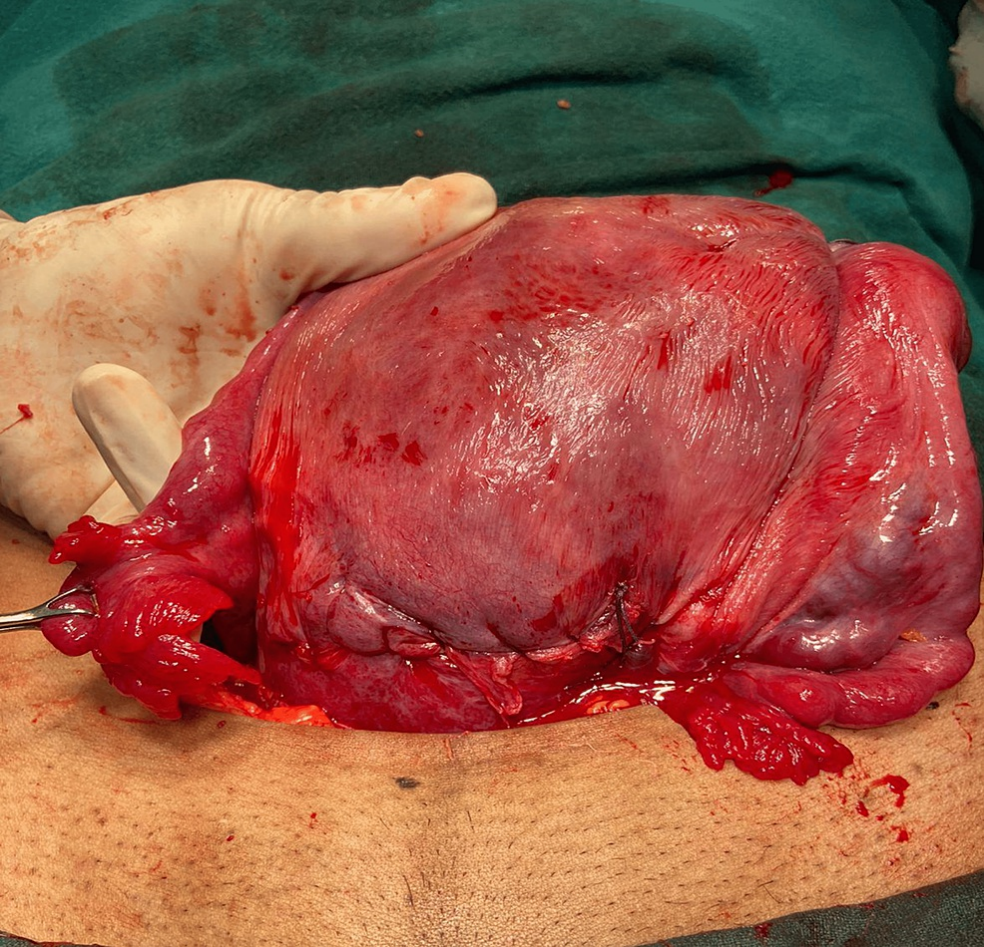
A noncavitary rudimentary horn of the uterus (like the fibromuscular band) with its well-formed fallopian tube and ovary separately on the left; the right-sided horn was well-developed Photo credit: author

She conceived spontaneously two and half years later after stopping every medication. Throughout her whole antenatal period, she had pain in the abdomen, off and on, relieved by antispasmodics and progesterone supplementation. At 34 weeks, she came with severe pain, breech presentation, and fetal bradycardia. An emergency LSCS was performed on her. While extraction of the baby by breech, the uterus was seen tubular and typically banana-shaped like a unicornuate uterus (Figure 1). After delivery of the baby, it was observed that the left-sided tube and ovary were not directly attached to the left cornu. Some fibromuscular structure was there in between the main uterus and the tubo-ovarian complex. After proper inspection, it was appreciated as a noncavitary rudimentary horn of the uterus with its well-formed fallopian tube and ovary separately (Figure 2). A male baby of 2.2 kg birthweight was delivered and kept for 48 hours of observation due to mild respiratory distress. Uterine suturing was done in two layers and the abdomen was closed in layers.

**Figure 3:**
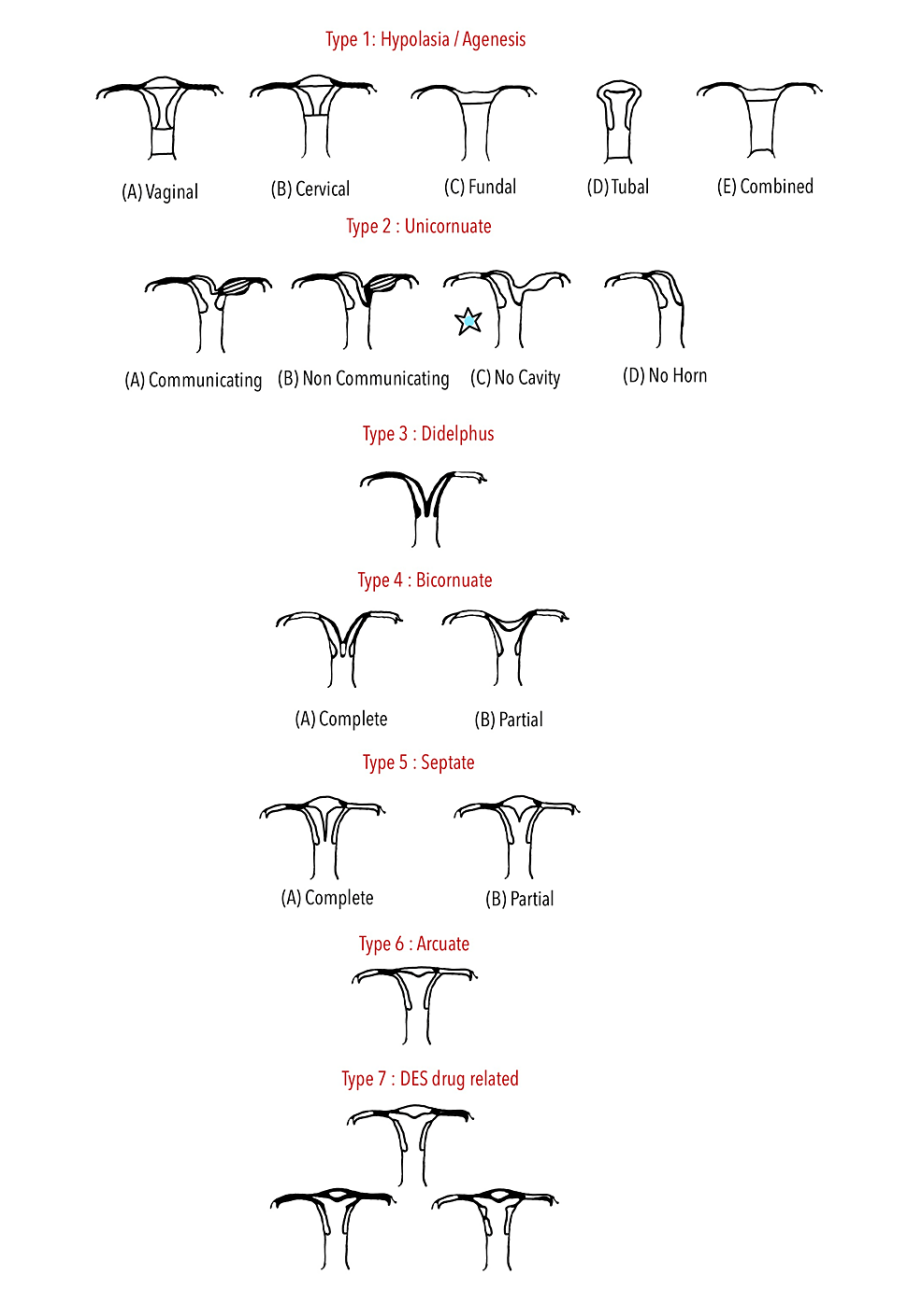
AFS's classification of Müllerian defects This figure is adapted from the AFS's classification of uterine anomalies [7]. AFS, American Fertility Society

Post-operative period was uneventful, and both mother and baby were discharged well in good condition. Post-operatively, her magnetic resonance imaging (MRI) was done to find out any associated renal malformation. When correlated with the American Fertility Society's (AFS) classification of Müllerian defects, it was found to have a resemblance with Type 2C kind of unicornuate uterus with solid rudimentary horn without endometrial lining and endometrial cavity, because of which she remained asymptomatic except for having chronic lower abdominal pain off and on throughout her pregnancy (Figure 3).

## Discussion

Birth defects of the female genital tract known as MDAs come in a variety of forms. Unicornuate uterine abnormalities are mentioned as the second subgroup in the American Society of Reproductive Medicine's classification that has been used for years [5]. 

A unicornuate uterus with a rudimentary horn (UUWRH) affects about one in 100,000 healthy women [6]. Only 14% of cases of pregnancy in a non-communicating rudimentary horn are appropriately detected before clinical symptoms appear, and the pregnancy incidence rate in these situations ranges from one in 76,000 to one in 50,000 [8].

The majority of abnormalities of the female biological system are congenital uterine deformities [1]. The physiological generation and change of the Müllerian ducts involve the processes of organogenesis, fusion, and septal resorption. The formation of the uterus, cervix, and fallopian tube begins with the disintegration of midline tissue in the 20th week of pregnancy [2]. Any of the aforementioned stages may impede the growth of these entities. Regression of the Müllerian ducts is brought on by the synthesis of the AMH and testosterone in males (46,XY). Because of this, in genetically female embryos (46,XX), the Müllerian ducts can develop into these aforementioned organs due to the absence of Y chromosomes [2].

The AFS has determined seven main categories of abnormalities [3]. The lack of one or both Müllerian ducts results from early organogenesis malfunction, which also results in uterine agenesis/hypoplasia or a unicornuate uterus. A unicornuate uterus with a crude horn is a result of canalization failure, and it also arises during the early stages of embryogenesis (seven to eight weeks of gestation). It is estimated that unicornuate uteri make up 2.5% to 13.7% of all uterovaginal abnormalities [8].

A total of 7-48% of cases of a unicornuate uterus allegedly had a non-communicating rudimentary uterine horn [9]. On average, the presence of a rudimentary horn is uncommon (0.06%). It was discovered that the rudimentary horn was linked to a poor prognosis for pregnancy as well as a high prevalence of cornual pregnancies, cystitis, and menstrual cramps.

The damaged anatomical structures are often excised or rebuilt laparoscopically as part of the treatment. The diagnosis and surgical treatment of this entity remains difficult because of its uncommon incidence. After extensive counseling, the individual treatment plan must be devised. In particular, case studies of the experimental therapy of Müllerian abnormalities paved the path for advancements in the surgical and diagnostic management of these conditions [9,10].

Occult Müllerian deformities are not uncommon, and they may be diagnosed whenever laparoscopy or laparotomy is performed due to some other indication. The association of renal anomalies always needs to be screened. Management may vary from no intervention to exhaustive repairs with a multidisciplinary approach depending on the number of combined defects. Sometimes no surgical correction is possible and the patient can only be counseled for future sexual and obstetric prospects [5,11].

## Conclusions

Müllerian anomalies may surprise us up to any extent from asymptomatic occult existence to very severe dysmenorrhea, abdominopelvic pain, infertility, and associated renal dysgenesis.

The present case could only be diagnosed intraoperatively. Although the patient suffered from infertility for a long period, she conceived spontaneously and fortunately did not have associations with any renal deformities. This type of scenario should be dealt with a high degree of suspicion.
